# Nutrition as prevention for improved cancer health outcomes: a systematic literature review

**DOI:** 10.1093/jncics/pkad035

**Published:** 2023-05-22

**Authors:** Helen M Parsons, Mary L Forte, Hamdi I Abdi, Sallee Brandt, Amy M Claussen, Timothy Wilt, Mark Klein, Elizabeth Ester, Adrienne Landsteiner, Aasma Shaukut, Shalamar S Sibley, Joanne Slavin, Catherine Sowerby, Weiwen Ng, Mary Butler

**Affiliations:** Minnesota Evidence-Based Practice Center, Division of Health Policy and Management, School of Public Health, University of Minnesota, Minneapolis, MN, USA; Minnesota Evidence-Based Practice Center, Division of Health Policy and Management, School of Public Health, University of Minnesota, Minneapolis, MN, USA; Minnesota Evidence-Based Practice Center, Division of Health Policy and Management, School of Public Health, University of Minnesota, Minneapolis, MN, USA; Minnesota Evidence-Based Practice Center, Division of Health Policy and Management, School of Public Health, University of Minnesota, Minneapolis, MN, USA; Minnesota Evidence-Based Practice Center, Division of Health Policy and Management, School of Public Health, University of Minnesota, Minneapolis, MN, USA; Minnesota Evidence-Based Practice Center, Division of Health Policy and Management, School of Public Health, University of Minnesota, Minneapolis, MN, USA; Minneapolis VA Center for Care Delivery and Outcomes Research, Minneapolis, MN, USA; School of Medicine, University of Minnesota, Minneapolis, MN, USA; Minneapolis VA Healthcare System, Minneapolis, MN, USA; School of Medicine, University of Minnesota, Minneapolis, MN, USA; Minneapolis VA Healthcare System, Minneapolis, MN, USA; University of Minnesota Physicians, Minneapolis, MN, USA; Minneapolis VA Center for Care Delivery and Outcomes Research, Minneapolis, MN, USA; New York University Langone, New York, NY, USA; School of Medicine, University of Minnesota, Minneapolis, MN, USA; Minneapolis VA Healthcare System, Minneapolis, MN, USA; Department of Food Science and Nutrition, College of Food, Agricultural and Natural Resource Sciences, St. Paul, MN, USA; Minneapolis VA Center for Care Delivery and Outcomes Research, Minneapolis, MN, USA; Minnesota Evidence-Based Practice Center, Division of Health Policy and Management, School of Public Health, University of Minnesota, Minneapolis, MN, USA; Minnesota Evidence-Based Practice Center, Division of Health Policy and Management, School of Public Health, University of Minnesota, Minneapolis, MN, USA

## Abstract

**Background:**

Among adults with cancer, malnutrition is associated with decreased treatment completion, more treatment harms and use of health care, and worse short-term survival. To inform the National Institutes of Health Pathways to Prevention workshop, “Nutrition as Prevention for Improved Cancer Health Outcomes,” this systematic review examined the evidence for the effectiveness of providing nutrition interventions before or during cancer therapy to improve outcomes of cancer treatment.

**Methods:**

We identified randomized controlled trials enrolling at least 50 participants published from 2000 through July 2022. We provide a detailed evidence map for included studies and grouped studies by broad intervention and cancer types. We conducted risk of bias (RoB) and qualitative descriptions of outcomes for intervention and cancer types with a larger volume of literature.

**Results:**

From 9798 unique references, 206 randomized controlled trials from 219 publications met the inclusion criteria. Studies primarily focused on nonvitamin or mineral dietary supplements, nutrition support, and route or timing of inpatient nutrition interventions for gastrointestinal or head and neck cancers. Most studies evaluated changes in body weight or composition, adverse events from cancer treatment, length of hospital stay, or quality of life. Few studies were conducted within the United States. Among intervention and cancer types with a high volume of literature (n = 114), 49% (n = 56) were assessed as high RoB. Higher-quality studies (low or medium RoB) reported mixed results on the effect of nutrition interventions across cancer and treatment-related outcomes.

**Conclusions:**

Methodological limitations of nutrition intervention studies surrounding cancer treatment impair translation of findings into clinical practice or guidelines.

Among adults with cancer, malnutrition is associated with decreased treatment completion, greater treatment-related adverse events and health-care use, and worse survival (1-4). Cancer-related malnutrition results from inadequate nutrition intake due to the systemic effects of the disease, adverse or negative effects of cancer and treatment, and other factors (5,6). Adults with cancer commonly experience malnutrition, with estimates ranging between 25% and 80% across patient populations (7-9). However, malnutrition substantially varies by patient characteristics, such as age at diagnosis, cancer type, stage of disease, type of cancer treatment, and preexisting conditions (eg, diabetes), among other factors (8,10). Further, many factors may increase risk or severity of malnutrition, including cancer location (head and neck or gastrointestinal), symptoms (eg, anorexia, early satiety, and fatigue), treatment modalities (surgery, chemo or radiotherapy), complications (eg, mucositis, nausea, taste changes), and psychological distress. In individuals with cancer, malnutrition often goes unrecognized by clinicians and patients and family or caregivers (11). Even when recognized, malnutrition may not be adequately addressed. Only 30% to 50% of cancer patients at risk for malnutrition receive nutrition intervention (12,13). Considering that an estimated 1.9 million individuals were diagnosed with cancer in 2021, between 570 000 and 950 000 individuals may have or be at risk for malnutrition.

Both the American Society for Parenteral and Enteral Nutrition and the American College of Surgeons Commission on Cancer recommend initial malnutrition screening and subsequent periodic reassessment during the course of cancer treatment and survivorship (6,14,15). However, no guidelines based on comprehensive, high-quality evidence exist for prevention, screening, or treatment of malnutrition in adults with cancer, creating challenges for individuals with cancer and their clinicians. Furthermore, we do not know how treatment benefits and harms may be affected by patient characteristics (eg, age, race and ethnicity, family or other support, socioeconomic status including food security, prediagnosis obesity, or sarcopenia), cancer-related factors (eg, cancer type, stage), treatment type (chemotherapy, radiation, surgery), treatment timing (before or after treatment), and provider, hospital, and geographic characteristics (eg, presence of integrated nutrition programs; specialist type, availability, and insurance coverage; and rural or urban location). Understanding the most effective interventions for nutrition in this population is critical, given the poor access to outpatient nutrition care for cancer patients across the United States (16).

To summarize the evidence, we conducted a systematic literature review that examined the effectiveness of nutrition interventions before or during cancer therapy to improve outcomes of cancer treatment. Additionally, we identified research gaps and challenges to inform expert and stakeholder discussions at the National Institutes of Health Pathways to Prevention workshop, “Nutrition as Prevention for Improved Cancer Health Outcomes,” which took place July 26-28, 2022. We also aimed to inform development of a research agenda for evaluating nutrition interventions in inpatient and outpatient cancer care in the United States. Our results can inform clinical guidelines on the prevention and treatment of malnutrition in cancer care.

## Methods

The review was guided by a set of key questions (KQ) and contextual questions (CQ), including:

- In adults diagnosed with cancer who have or are at risk for cancer-associated malnutrition, what is the effect of nutrition interventions before (KQ 1) or during (KQ 2) cancer treatment in preventing negative treatment outcomes such as effects on dose tolerance, hospital use, adverse events, and survival?
- What is the effect of nutrition interventions before or during cancer treatment on associated symptoms such as fatigue, nausea and vomiting, appetite, physical and functional status (eg, frailty), and quality of life (KQ 3)?
- In adults with cancer who are overweight or obese, what is the effect of nutrition interventions intended for weight loss before or during cancer treatment in preventing negative treatment outcomes such as effects on dose, hospital use, adverse events, and survival (KQ 4)?
- What evidence is available on the cost-effectiveness of nutrition interventions for preventing negative outcomes associated with cancer treatment (CQ)?

For KQs 1-3, we further examined the evidence on variation in effects of nutrition interventions 1) by cancer type, treatment type, and stage of disease; 2) across the lifespan (eg, adults aged ≥65 years vs <65 years); 3) in adults with or without muscle wasting; and 4) across special populations (eg, individuals with multiple comorbid conditions).

We conducted a comprehensive literature search in July 2022, searching MEDLINE (Ovid), Embase (Ovid), and Cochrane Central Register of Controlled Trials (Wiley) for studies that evaluated a broad range of nutrition interventions (eg, dietary supplements, nutrition support, nutrition counseling) for preventing and treating negative outcomes of cancer and its treatment (see Supplementary Materials, available online for our complete search strategy). The search included literature published from 2000 through July 2022 to encompass contemporary cancer treatments. We included studies that met our prespecified criteria outlined in Table 1. We further limited our search to randomized controlled trials (RCTs) published in English in a peer-reviewed journal. To identify the literature with the highest likelihood of having statistical power to detect an effect from a nutrition intervention, we limited included studies to those randomly assigning at least 50 participants (ie, approximately 25 individuals per arm). Studies that described the cost or value (eg, cost-effectiveness, cost-benefit) of nutrition interventions were eligible for inclusion in the CQ.

**Table 1.** Population, intervention, comparator, outcome, timing, and setting (PICOTS) for included studies

KQ	KQ1	KQ2	KQ3	KQ4
Population	Adults diagnosed with cancer at or after age 18 y who have or are at risk for cancer-associated malnutrition Subgroups: Cancer and treatment characteristics (cancer type, treatment type (systemic therapy, radiation, surgery), stage of disease) Adults ≥65 y vs younger Muscle wasting (eg, sarcopenia, cachexia, precachexia) vs no muscle wasting Special populations (individuals with multiple comorbid conditions)	Adults diagnosed with cancer at or after age 18 y who have or are at risk for cancer-associated malnutrition Subgroups: Cancer and treatment characteristics (cancer type, treatment type (systemic therapy, radiation, surgery), stage of disease) Adults ≥65 y vs younger Muscle wasting (eg, sarcopenia, cachexia, precachexia) vs no muscle wasting Special populations (individuals with multiple comorbid conditions)	Adults diagnosed with cancer at or after age 18 y who have or are at risk for cancer-associated malnutrition Subgroups: Cancer and treatment characteristics (cancer type, treatment type (systemic therapy, radiation, surgery), stage of disease) Adults ≥65 y vs younger Muscle wasting (eg, sarcopenia, cachexia, precachexia) vs no muscle wasting Special populations (individuals with multiple comorbid conditions)	Overweight (BMI 25 to <30)/obese (BMI ≥30) adults aged ≥18 y diagnosed with cancer
Intervention	Nutrition interventions under supervision of nutrition professional (eg, dietitian, nutritionist, or other licensed clinicians) Diet or nutrition therapy (via oral or enteral (eg, nasogastric, gastrostomy, jejunostomy) feeding Special diets (eg, fasting (intermittent or short term), calorie restriction, ketogenic, Mediterranean diet, high calorie, high protein) Supplements, nonvitamin/mineral Total parenteral therapy Nutrition counseling Combined nutrition interventions (eg, nutrition counseling with nutrition therapy)	Nutrition interventions under supervision of nutrition professional (eg, dietitian, nutritionist, or other licensed clinicians) Diet or nutrition therapy (via oral or enteral (eg, nasogastric, gastrostomy, jejunostomy) feeding Special diets (eg, fasting (intermittent or short term), calorie restriction, ketogenic, Mediterranean diet, high calorie, high protein) Supplements, nonvitamin/mineral Total parenteral therapy Nutrition counseling Combined nutrition interventions (eg, nutrition counseling with nutrition therapy)	Nutrition interventions under supervision of nutrition professional (eg, dietitian, nutritionist, or other licensed clinicians) Diet or nutrition therapy (via oral or enteral (eg, nasogastric, gastrostomy, jejunostomy) feeding Special diets (eg, fasting (intermittent or short term), calorie restriction, ketogenic, Mediterranean diet, high calorie, high protein) Supplements, nonvitamin/mineral Total parenteral therapy Nutrition counseling Combined nutrition interventions (eg, nutrition counseling with nutrition therapy)	Nutrition interventions intended for weight loss (includes both PNIs and NIDTs)
Comparators	Standard of care vs PNIs or PNIs vs PNIs	Standard of care vs NIDTs, NIDT vs NIDT or PNIs vs NIDTs	Standard of care vs PNIs or NIDTs, NIDTs vs NIDTs, PNIs vs PNIs, PNIs vs NIDTs	Standard of care vs PNIs or NIDTs, NIDTs vs NIDTs, PNIs vs PNIs, PNIs vs NIDTs
Outcomes	Intermediate outcomes BMI, body composition, weight (loss, gain) Final outcomes Cancer treatment tolerance: treatment interruptions, reductions, or delays Hospital use: ER visits, admissions, length of hospital stay Adverse events Chemotherapy/radiation therapy limiting toxicity Postop complication NI-related AEs Unintended harms Survival Nutrition status Malnutrition (underweight, wasting, overweight)	Intermediate outcomes BMI, body composition, weight (loss, gain) Final outcomes Cancer treatment tolerance: treatment interruptions, reductions, or delays Hospital use: ER visits, admissions, length of hospital stay Adverse events Chemotherapy/radiation therapy limiting toxicity Postop complication NI-related AEs Unintended harms Survival Nutrition status Malnutrition (underweight, wasting, overweight)	Fatigue, nausea and vomiting, appetite, physical/functional status (eg, frailty) Quality of life	Intermediate outcomes BMI, body composition, weight (loss, gain) Final outcomes Cancer treatment tolerance: treatment interruptions, reductions, or delays Hospital use: ER visits Admissions, length of hospital stay Adverse events Chemotherapy/radiation therapy limiting toxicity Postop complication NI-related AEs Unintended harms Survival Nutrition status Malnutrition (underweight, wasting, overweight)
Timing	Nutrition interventions delivered precancer treatment (KQ1, KQ3, KQ4) and during cancer treatment (KQ2, KQ3, KQ4)	Nutrition interventions delivered precancer treatment (KQ1, KQ3, KQ4) and during cancer treatment (KQ2, KQ3, KQ4)	Nutrition interventions delivered precancer treatment (KQ1, KQ3, KQ4) and during cancer treatment (KQ2, KQ3, KQ4)	Nutrition interventions delivered precancer treatment (KQ1, KQ3, KQ4) and during cancer treatment (KQ2, KQ3, KQ4)
Setting	Outpatient oncology care, ambulatory care, cancer treatment centers, inpatient, home based, hospice, telemedicine	Outpatient oncology care, ambulatory care, cancer treatment centers, inpatient, home based, hospice, telemedicine	Outpatient oncology care, ambulatory care, cancer treatment centers, inpatient, home based, hospice, telemedicine	Outpatient oncology care, ambulatory care, cancer treatment centers, inpatient, home based, hospice, telemedicine

AE = adverse event; BMI = body mass index; ER = emergency room; KQ = key question; NI = nutrition intervention; NIDT = nutrition intervention during treatment; PNI = pretreatment nutrition intervention; RCT = randomized controlled trial.

To identify eligible studies, search results were downloaded to PICO Portal (17), an online systematic review platform, for screening. Two trained, independent investigators reviewed titles and abstracts for identified studies meeting population, intervention, comparator, outcome, timing, and setting framework and study selection criteria. Two reviewers independently performed full-text screening to determine whether studies met inclusion criteria. Differences in screening decisions were resolved by consultation between reviewers, and, if necessary, consultation with a third investigator. All citations deemed appropriate for inclusion through title and abstract review by both reviewers were then examined in the full text. We documented inclusion and exclusion status of citations, noting reasons for exclusion. Throughout the screening process, members of the review team regularly met to discuss training material and issues as they arose to ensure that inclusion criteria were consistently applied.

Among studies deemed eligible for inclusion in one of the KQs, 2 independent reviewers assessed risk of bias (RoB) on a subset of eligible studies based on Agency for Healthcare Research and Quality guidance. This subset of studies included those that had a relatively large number of studies within an intervention and cancer type. We used a threshold of 10 studies within a specific intervention or cancer type; dietary supplements and nutrition support for gastrointestinal cancers were frequent categories for RoB assessment, which included all studies of dietary supplements and nutrition support for gastrointestinal cancers across KQs. Any discrepancies in overall RoB assessments were resolved through discussion. We classified overall RoB for each study as low, moderate, or high based on the collective RoB inherent in each domain and confidence that the results are believable given the study’s limitations.

We then extracted basic study information from all eligible studies that met the inclusion criteria. Among studies for which RoB was assessed, we further abstracted information on intervention duration, comparisons, and outcomes for those studies deemed to have low or medium RoB. Meta-analysis was generally not feasible or appropriate because of the considerable heterogeneity of intervention types, comparators, outcomes, and timing evaluated within KQs. We organized the results by KQ then broadly by type of nutrition intervention and type of cancer. Given the lack of recognized classification systems for grouping or describing nonpharmacologic nutrition interventions, we grouped studies into single intervention types based on the content and intent of the intervention and the intended audience, using study author-supplied taxonomies and definitions where available (Table 2).

**Table 2.** Systematic review intervention categories and descriptions

Intervention category	Description
Nutrition counseling	Nutrition counseling involves an individualized nutrition assessment followed by personalized care of nutrition and diet-related needs with the goal of achieving and maintaining optimal nutrition status across the continuum of care.
Dietary supplements	Dietary supplements include products (eg, added arginine, glutamine, fish oil) containing 1 or more ingredients meant to supplement the diet for improved nutrition status but not meant to replace calories. Vitamins, minerals, and antioxidants were not included.
Special diets	Special diets include the use of defined nutrition plans or approaches such as fasting (intermittent or short term), calorie restriction, ketogenic, Mediterranean, high-calorie, high-protein diets to support cancer care, and others.
Route or timing of nutrition interventions	Route or timing interventions involve testing only the route (eg, percutaneous endoscopic gastrostomy [PEG] tube, enteral feeding) or timing (eg, initiation or duration) of nutrition interventions with similar nutrition contents.
Nutrition support including oral nutrition supplements	Nutrition support interventions involve the use of parenteral nutrition, enteral nutrition (tube feeding), and oral nutrition supplements (eg, Ensure, Boost, immunonutrition [oral nutrition supplements that include a set of nutrients meant to have an effect of the immune system]) (240) to maintain or improve nutrition status.
Multi-component interventions	Multi-component interventions involve multiple strategies for nutrition interventions, such as counseling plus use of dietary supplements.

The final protocol was posted online on October 8, 2021 (https://effectivehealthcare.ahrq.gov/products/improved-cancer-outcomes/protocol). We registered the protocol on PROSPERO (CRD42021282881). Additional review details, including our RoB assessment tool, can be found as part of the full systematic review posted on the Agency for Healthcare Research and Quality and Pathways to Prevention websites.

## Results

**Figure 1.**
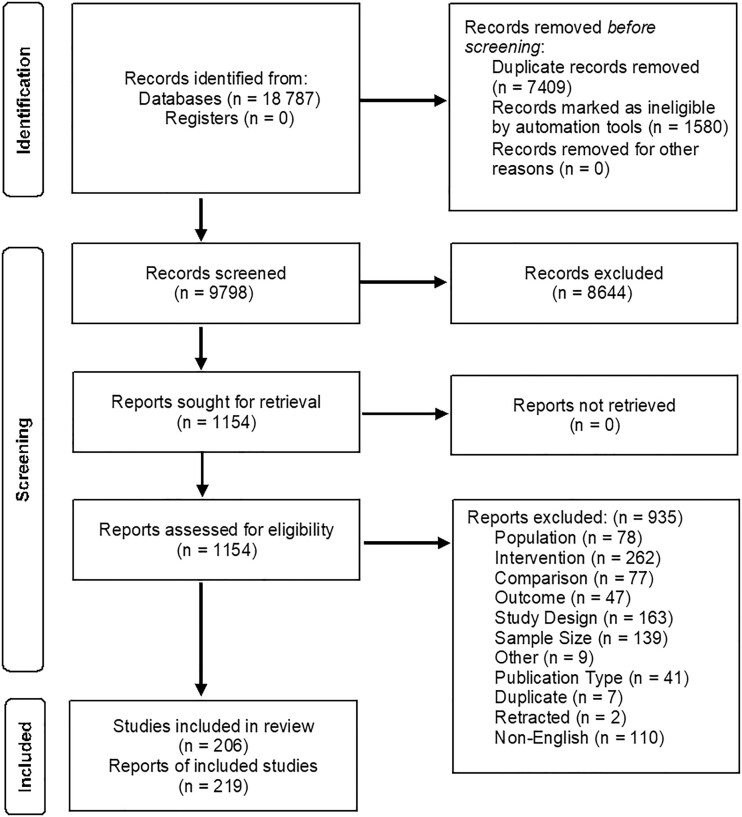
Preferred Reporting Items for Systematic Reviews and Meta-analyses (PRISMA) diagram.

**Figure 2.**
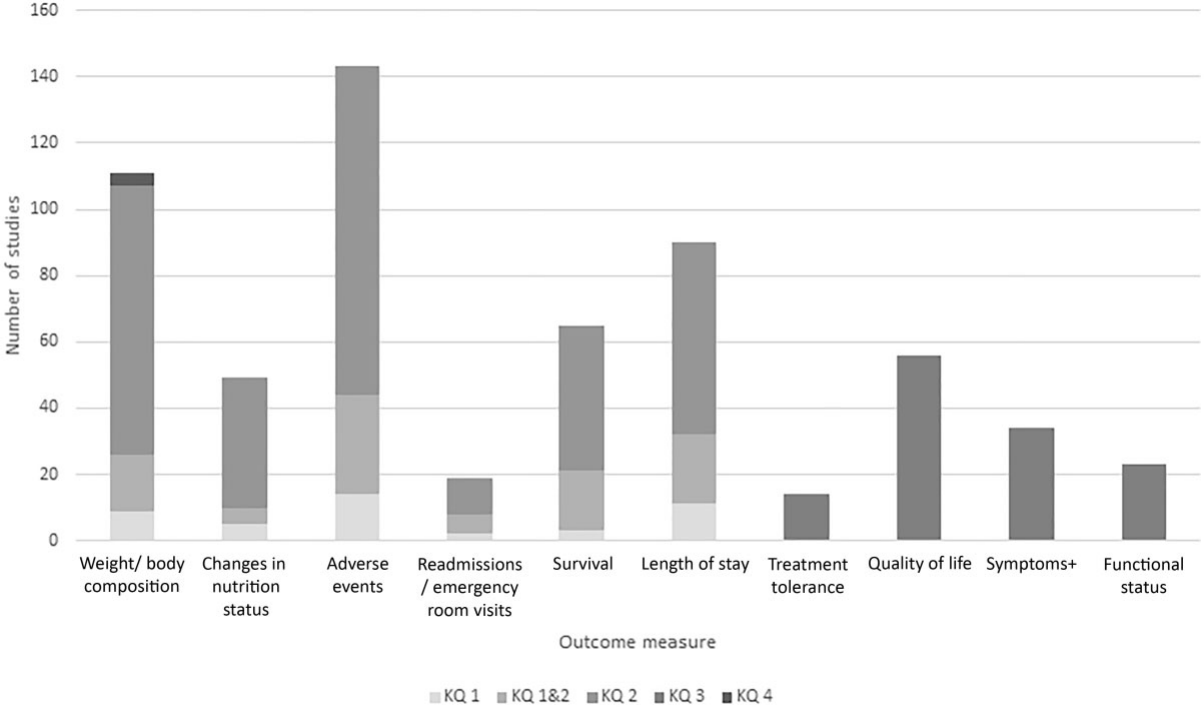
Number of studies evaluating outcomes by key question (KQ), N = 206. Studies may evaluate more than 1 outcome; + Symptoms may include cancer or cancer treatment related symptoms such as fatigue, nausea and vomiting, appetite. KQs: In adults diagnosed with cancer who have or are at risk for cancer-associated malnutrition: What is the effect of nutrition interventions before (KQ1) or during (KQ2) cancer treatment in preventing negative treatment outcomes such as effects on dose tolerance, hospital use, adverse events, and survival? What is the effect of nutrition interventions before or during cancer treatment on associated symptoms such as fatigue, nausea and vomiting, appetite, physical and functional status (eg, frailty), and quality of life (KQ3)? In adults with cancer who are overweight or obese, what is the effect of nutrition interventions before or during cancer treatment in preventing negative treatment outcomes such as effects on dose, hospital use, adverse events and survival (KQ4)?

From 9798 unique references, we identified 206 RCTs described in 219 publications on nutrition interventions to improve cancer treatment outcomes that met inclusion criteria (Figure 1). The randomized trial evidence on nutrition interventions for adults before and/or during cancer treatment was extremely broad and focused on dietary supplements, nutrition support (enteral and parenteral nutrition, including oral nutrition supplements), and the route or timing of nutrition interventions (Table 3). Studies were predominantly conducted in populations with gastrointestinal and head and neck cancers. Less than one-half of enrollees had American Joint Commission on Cancer Stage IV disease among studies reporting tumor stage. Studies included both inpatient surgical and outpatient settings (Table 3) and focused on evaluating changes in body weight or composition, treatment-related adverse events, length of hospital stay, and quality of life (Figure 2). Most studies included patients considered “at risk” for (eg, gastrointestinal cancers, receiving inpatient surgical treatment, advanced disease) malnutrition (Table 4). A variety of malnourishment screening tools were used, with many studies not reporting malnourishment assessment method. Few studies were conducted within the US setting. Among studies with a high volume of literature, which predominately included studies of dietary supplements and nutrition support in gastrointestinal and head and neck cancers, 11% (n = 12) were rated as having low RoB (higher quality), 40% (n = 46) medium RoB, and 49% (n = 56) high RoB (low quality) (Table 3). Overall, studies widely differed in type of nutrition interventions administered, route and/or dose of administration, and control populations, even within KQ and intervention categories.

**Table 3.** Included studies by nutrition intervention and cancer type across key questions (KQ) (N = 206)^a^

KQ	Intervention	Head and neck cancer	Gastrointestinal cancer	Multiple cancers	Other cancer types^b^	Total^c^
Nutrition interventions before cancer treatment (KQ1)	Nutrition counseling	0	0	0	0	0
	Dietary supplements	1	2^c^ 2 High RoB	0	2	5
	Special diets	0	0	0	0	0
	Route or timing of nutrition interventions	0	0	0	0	0
	Nutrition support including oral nutrition supplements	0	15^c^ 2 Low RoB 6 Medium RoB 7 High RoB	0	0	15
	Multi-component interventions	0	0	0	0	0
Total						20
Nutrition interventions before and including initiation of cancer treatment (spans KQ 1 and 2)	Nutrition counseling	0	0	0	0	0
	Dietary supplements	2	10^c^ 4 Medium RoB 6 High RoB	2	1	15
	Special diets	0	0	0	0	0
	Route or timing of nutrition interventions	2	6	1	0	9
	Nutrition support including oral nutrition supplements	1	10^c^ 2 Low RoB 4 Medium RoB 4 High RoB	0	2	13
	Multi-component interventions	0	0	1	0	1
Total						38
Nutrition interventions after treatment began (KQ2)	Nutrition counseling	3	4	6	2	15
	Dietary supplements	10^c^ 6 Medium RoB 4 High RoB	16^c^ 4 Low RoB 8 Medium RoB 4 High RoB	3	5	34
	Special diets	0	1	2	5	8
	Route or timing of nutrition interventions	4	24^c^ 1 Low RoB 10 Medium RoB 13 High RoB	1	2	31
	Nutrition support including oral nutrition supplements	4	27^c^ 3 Low RoB 8 Medium RoB 16 High RoB	4	8	43
	Multi-component interventions	2	2	2	4	10
Total						141
Effect of nutrition interventions on symptoms (KQ3)	Nutrition counseling	1	4	4	3	12
	Dietary supplements	1	5^c^ 3 Medium RoB 2 High RoB	3	1	10
	Special diets	0	1	2	7	10
	Route or timing of nutrition interventions	2	4	1	1	8
	Nutrition support including oral nutrition supplements	5	20^c^ 5 Low RoB 4 Medium RoB 11 High RoB	3	4	32
	Multi-component interventions	2	0	3	2	7
Total						79
Effect of nutrition Interventions on weight loss (KQ4)	Nutrition counseling	0	0	0	0	0
	Dietary supplements	0	0	0	0	0
	Special diets	0	0	0	4	4
	Route or timing of nutrition interventions	0	0	0	0	0
	Nutrition support including oral nutrition supplements	0	0	0	0	0
	Multi-component interventions	0	0	0	0	0
Total						4

a Totals are not mutually exclusive to number of included studies. Studies addressing KQ3 could also address KQ1, KQ2, or span KQ1 and 2. RoB = risk of bias.

b Other cancer types include studies evaluating all remaining cancer types not included in previous categories (eg, breast, prostate, lung cancer). Includes a total of 206 unique studies across 219 publications. KQs: In adults diagnosed with cancer who have or are at risk for cancer-associated malnutrition: What is the effect of nutrition interventions before (KQ1) or during (KQ2) cancer treatment in preventing negative treatment outcomes such as effects on dose tolerance, hospital use, adverse events, and survival? What is the effect of nutrition interventions before or during cancer treatment on associated symptoms such as fatigue, nausea and vomiting, appetite, physical and functional status (eg, frailty), and quality of life (KQ3)? In adults with cancer who are overweight or obese, what is the effect of nutrition interventions before or during cancer treatment in preventing negative treatment outcomes such as effects on dose, hospital use, adverse events, and survival (KQ4)?

c Indicates studies in which RoB was assessed.

**Table 4.** Characteristics of included studies examining the impact of nutrition interventions before or during cancer treatment (N = 206)

Characteristics	KQ1	Spans KQ1 and 2	KQ2	KQ3	KQ4
Total included studies^a^	20 Studies	38 Studies	141 studies	79 studies	4 studies
Intervention type	5 Dietary supplements 15 Nutrition support including oral nutrition supplements	15 Dietary supplements 9 Route or timing of nutrition interventions 13 Nutrition support including oral nutrition supplements 1 Multi-component interventions	15 Nutrition counseling 34 Dietary supplements 8 Special diets 31 Route or timing of nutrition interventions 43 Nutrition support including oral nutrition supplements 10 Multi-component interventions	12 Nutrition counseling 10 Dietary supplements 10 Special diets 8 Route or timing of nutrition interventions 32 Nutrition support including oral nutrition supplements 7 Multi-component interventions	4 Special diets
Study sample size	9 50-75 3 76-100 8 > 100	9 50-75 7 76-100 22 > 100	37 50-75 38 76-100 66 > 100	13 50-75 25 76-100 41 > 100	2 76-100 2 > 100
Cancer type	1 Head and neck 17 Gastrointestinal 2 Other cancer types^b^	5 Head and neck 26 Gastrointestinal 4 Multiple cancers 3 Other cancer types^b^	23 Head and neck 74 Gastrointestinal 18 Multiple 26 Other cancer types^b^	11Head and neck 34 Gastrointestinal 16 Multiple 18 Other cancer types^b^	4 Other
Intervention delivery setting	12 Inpatient 4 Outpatient 1 Multiple settings 1 Other 2 Not reported	20 Inpatient 6 Outpatient 6 Multiple settings 1 Other 5 Not reported	70 Inpatient 44 Outpatient 5 Multiple settings 5 Other 17 Not reported	25 Inpatient 36 Outpatient 4 Multiple settings 6 Other 8 Not reported	3 Outpatient 1 Other
Dominant cancer treatment type of participants	16 Surgery alone 2 Chemotherapy alone 2 Multiple therapies	30 Surgery alone 4 Chemotherapy alone 1 Radiation alone 3 Multiple therapies	67 Surgery alone 24 Chemotherapy alone 12 Radiation alone 34 Multiple therapies 4 Not reported	24 Surgery alone 19 Chemotherapy alone 11 Radiation alone 23 Multiple therapies 2 Not reported	2 Chemotherapy 2 Multiple therapies
Limited to malnourished patients	1 Yes 19 No	10 Yes 27 No 1 Not reported	22 Yes 118 No 1 Not reported	15 Yes 63 No 1 Not reported	2 No
Malnourishment screening tool used	5 Nutrition risk screening NRS-2002 7 Other tool/metric 1 Multiple tools/metrics 7 Not reported	3 Nutrition risk screening-2002 2 Malnourishment Screening Tool 14 Other tool/metric 2 Multiple tools/metrics 17 Not reported	18 Nutrition risk screening NRS-2002 3 Mini Nutritional Assessment (MNA) or MNA-Short Form (SF) 1 Malnutrition Universal Screening Tool (MUST) 30 Other tool/metric 10 Multiple tools 79 Not reported	13 Nutrition risk screening NRS-2002 1 MUST 19 Other tool/metric 6 Multiple tools 40 Not reported	4 Not reported
Provider prescribing or delivering the intervention	5 Dietitian/nutritionist 1 Physician 1 Other 1 Multiple 12 Not reported	6 Dietitian/nutritionist 1 Nurse 1 Physician 2 Other 3 Multiple providers 25 Not reported	25 Dietitian/nutritionist 4 Nurse 15 Physician 2 Other 22 Multiple 73 Not reported	26 Dietitian/nutritionist 2 Nurse 4 Physician 2 Other 15 Multiple 30 Not reported	2 Dietitian/nutritionist 1 Multiple 1 Not reported
Route of administration	15 Oral 2 Enteral 2 Parenteral 1 Not reported	20 Oral 10 Enteral 4 Parenteral 3 Other 1 Not reported	61 Oral 37 Enteral 15 Parenteral 12 Other 1 Not reported 15 Not applicable	47 Oral 10 Enteral 7 Parenteral 3 Other 12 Not applicable	4 Oral
Geographic region of intervention	9 Asia 8 Europe 1 North America 2 Other	16 Asia 17 Europe 2 North America 3 Other	75 Asia 49 Europe 6 North America 10 Other 1 Not reported	37 Asia 26 Europe 6 North America 9 Other 1 Not reported	2 Asia 2 Europe
No, % of participants with stage IV disease	9, 0-25 1, >50 10 Not reported	10, 0-25 3, 26-50 3, >50 22 Not reported	50, 0-25 10, 26-50 15, 51-75 6, 76-100 60 Not reported	21, 0-25 6, 26-50 8, 51-75 8, 76-100 36 Not reported	4, 0-25
No, % female participants	4, 0-25 13, 26-50 2, 51-75 1, 76-100	10, 0-25 23, 26-50 3, 51-75 1, 76-100 1 Not reported	35, 0-25 67, 26-50 13, 51-75 13, 76-100 13 Not reported	17, 0-25 36, 26-50 9, 51-75 11, 76-100 6 Not reported	4, 76-100
Mean age of participants	12, 50-64 6, 65+ 2 Not reported	22, 50-64 13 ≥ 65 3 Not reported	9, <50 72, 50-64 36, ≥65 24 Not reported	4, <50 42, 50-64 22, ≥65 11 Not reported	4, 50-64
Outcomes evaluated^b^	9 Weight or body composition changes 5 Changes in nutrition status 14 Adverse events 2 Readmissions or emergency room visits 3 Survival 11 Length of stay	17 Weight or body composition changes 5 Changes in nutrition status 30 Adverse events 6 Readmissions or emergency room visits 18 Survival 21 Length of stay	81 Weight or body composition changes 39 Changes in nutrition status 99 Adverse events 11 Readmission or emergency room visits 44 Survival 58 Length of stay	14 Treatment tolerance 56 Quality of life 35 Symptoms 23 Functional status	4 Weight/body composition changes

a Totals are not mutually exclusive to number of included studies. Studies addressing key question (KQ)3 could also address KQ1, KQ2, or span KQ1 and 2.

b Studies may evaluate multiple outcomes: KQs: In adults diagnosed with cancer who have or are at risk for cancer-associated malnutrition: What is the effect of nutrition interventions before (KQ1) or during (KQ2) cancer treatment in preventing negative treatment outcomes such as effects on dose tolerance, hospital use, adverse events, and survival? What is the effect of nutrition interventions before or during cancer treatment on associated symptoms such as fatigue, nausea and vomiting, appetite, physical and functional status (eg, frailty), and quality of life (KQ3)? In adults with cancer who are overweight or obese, what is the effect of nutrition interventions before or during cancer treatment in preventing negative treatment outcomes such as effects on dose, hospital use, adverse events, and survival (KQ4)?

## Nutrition interventions before cancer treatment

For KQ1, we identified 20 unique studies that examined nutrition interventions before the initiation of cancer treatment. Studies examined the use of dietary supplements (n = 5) and nutrition support (n = 15). Table 3 includes basic characteristics of all included studies.

### Dietary supplements

We identified 5 unique studies examining dietary supplements used before cancer treatment (Table 4) (18-22). Studies most commonly evaluated outcomes including body weight or composition changes, adverse events, and survival. One study compared parenteral fish oil lipid emulsion (19), and another evaluated omega-3 fatty acids (20). Two studies evaluated the use of arginine, omega-3 fatty acids, and nucleotides in lung (21) and head and neck cancer patients (18). A final study evaluated preoperative oral carbohydrate loading before breast cancer surgery (22). Two studies were conducted in Brazil (19,20), 1 in Norway (22), 1 in Thailand (18), and 1 in Turkey (21).

Both studies evaluated for RoB were assessed as high RoB (low quality, Table 3), and outcomes were not evaluated (19,20).

### Nutrition support

We identified 15 unique studies examining nutrition support interventions before cancer treatment (Table 4) (23-37). Studies most commonly evaluated adverse events, length of stay, and body weight or composition changes.

Most interventions were preoperatively delivered in a surgical setting. Two studies evaluated daily oral supplementation with Fortisip oral nutrition support (23,24). Three studies examined preoperative use of immunonutrition, including use of oral immunonutrition in advanced pancreatic adenocarcinoma (31), enteral immunonutrition among patients with colorectal or gastric carcinoma (36), and immunonutrient-enriched supplementation in patients with colon cancer. Seven studies examined the use of more diverse oral nutrition support across gastrointestinal cancers, including use of preoperative Nutricia (35), hypercaloric supplementation (Nutridrink Protein) (28), peripheral intravenous nutrition for patients undergoing workup for biliopancreatic masses (29), oral nutrition combined with microbial preparations (33), preoperative oral supplementation for colorectal (34) and gastrointestinal cancers (27), and the use of Nutrison Fiber in patients with adenocarcinomas (37). The final 3 studies examined carbohydrate-rich beverages (26,32) as well as the use of 10% glucose solution before radical gastrectomy (25).

Among the 15 studies assessed for RoB (Table 3), 8 were assessed as low or medium RoB and reported mixed results on development of complications, improvements in weight loss, and length of hospital stay (Table 5) (23-25,27,30,32,34,36).

**Table 5.** Outcomes reported for low- and medium- risk-of-bias studies by key question (KQ) and intervention type^a^

KQ	Intervention type	RoB	No. range	Weight/body comp.	Changes in nutrition status	Adverse events	Readmissions/emergency room visits	Survival	LOS	Treatment tolerance	QoL	Symptoms	Functional status
Nutrition interventions before cancer treatment (KQ1)	Nutrition support including oral nutrition supplements	2 Low (27,32) 6 Medium (23-25,30,34,36)	50-161	2 ↑ (23,30) 2 ↔ (30,34)	1 ↔ (23)	2 ↑ (23,36) 6 ↔ (23-25,27,30,34)	2 ↔ (25,27)	1 ↔ (23)	2 ↑ (32,36) 4 ↔ (23,25,30,34)	—	—	—	—
Nutrition interventions before and including initiation of cancer treatment (spans KQ 1 and 2)	Dietary supplements	4 Medium (44,47-50)	60-195	1 ↑ (44) 2 ↔ (44,50)	—	4 ↔ (44,47-50)	2 ↔ (47-49)	4 ↔ (44,47-50)	4 ↔ (44,47-50)	—	—	—	—
	Nutrition support including oral nutrition supplements	2 Low (65,70) 4 Medium (63,64,71,74,75)	120-317	—	—	5 ↑ (63,64,70,71,74,75) 3 ↔ (65,71,74,75)	1 ↑ (65) 1 ↔ (71)	1 ↑ (63) 1 ↔ (71)	1 ↓ (63) 4 ↔ (64,70,71,74,75)	—	—	—	—
Nutrition interventions after treatment began (KQ2)	Dietary supplements	4 Low (109,111,123,129) 14 Medium (100-103,105,107,113,115-119,124,126)	50-229	3 ↑ (115-117) 10 ↔ (100-103,107,109,115,117-119)	1 ↔ (117)	11 ↑ (100,102,103,105,109,113,115,116,118,126,129) 13 ↔ (100-103,105,107,109,111,113,115-118,123,124,126,129)	1 ↑ (116) 1 ↔ (126)	6 ↔ (105,111,118,119,123,126)	5 ↑ (102,105,109,124,129) 6 ↔ (100,103,111,119,123,126)	—	—	—	—
	Route or timing of nutrition interventions	1 Low (141) 10 Medium (139,142,144,149,153,162,163,165-167)	60-317	2 ↑ (163,166) 4 ↔ (139,149,162,166)	1 ↓ (139)	4 ↑ (139,141,166,167) 1 ↓ (163) 7 ↔ (139,142,144,153,162,163,165)	1 ↔ (139)	5 ↔ (139,142,144,162,163)	5 ↑ (141,153,162,165,166) 4 ↔ (139,142,144,167)	—	—	—	—
	Nutrition support including oral nutrition supplements	3 Low (188-190,197,198,217) 8 Medium (172,177,191,192,194,199,211,212,214,217)	77-1003	5 ↑ (197,199,211,212,217) 3 ↔ (199,211,217)	1 ↑ (177)	4 ↑ (172,177,194,214) 6 ↔ (188-192,199,211,212,217)	2 ↔ (172,212)	1 ↑ (194) 3 ↔ (188-192,212)	3 ↑ (172,177,214) 3 ↔ (189,192,211,212)	—	—	—	—
Effect of nutrition interventions on symptoms (KQ3)	Dietary supplements	3 Medium (47,107,124)	71-229	—	—	—	—	—	—	—	1 ↑ (107) 1 ↔ (47)	1 ↑ (107) 1 ↔ (124)	—
	Nutrition support including oral nutrition supplements	5 Low (27,32,65,197,198,217) 4 Medium (25,34,211,212)	50-353	—	—	—	—	—	—	1 ↑ (197,198)	2 ↔ (212,217)	3 ↑ (32,65,197,198) 3 ↔ (25,27,32)	1 ↑ (32) 2 ↔ (34,217)

a Studies that reported statistically significant results for 1 aspect of an outcome and not statistically significant in another aspect of an outcome were recorded as separate instances. ↑ = Intervention group had a statistically significantly better outcome than comparison group (eg, fewer AEs, shorter LOS than comparison group); ↓ = intervention group had a statistically significantly worse outcome than comparison group (eg, more AEs, longer LOS); ↔ = no statistically significant difference between groups; AEs = adverse events; LOS = length of stay; QoL = quality of life; RoB = risk of bias.

## Nutrition interventions before and including initiation of cancer treatment

We identified 38 unique studies across 42 publications that examined nutrition interventions conducted both before and after initiation of cancer treatment. Studies examined the use of dietary supplements (n = 15), route or timing of nutrition interventions (n = 9), use of nutrition support (n = 13), and multicomponent interventions (n = 1). Table 3 includes basic characteristics of all included studies.

### Dietary supplements

We identified 15 unique studies across 16 publications examining the use of dietary supplements before and continued after the initiation of cancer treatment (Table 4) (38-53). Studies most commonly evaluated outcomes including adverse events, body weight or composition changes, survival, and length of stay. One group of studies examined the use of a single supplement for immunonutrition (eicosapentaenoic—[EPA], omega-3 fatty acid, or glutamine-enriched nutrition), but interventions varied by population and had a wide range of intervention doses and durations around the time of cancer surgery (42-46,48-52). The second group of studies examined use of supplements for immunonutrition, including a combination of L-arginine, omega-3 fatty acids, and nucleotides, a combination of arginine, nucleotides, and fatty acids, and immunomodulating nutrition (Oral Impact) relative to standard nutrition (38,40,53). The remaining studies evaluated a variety of other supplement combinations, including nutrition counseling with oral whey protein supplementation (39), omega-3 fatty acid and vitamin D (41), and perioperative oral protein supplementation rich in arginine and omega-6 (47). Studies were primarily conducted in Europe and Asia.

Among the 10 studies assessed for RoB (Table 3), 4 medium-RoB studies across 5 publications showed mixed results for the effect of dietary supplements on weight changes, readmissions, length of hospital stay, development of complications, and survival (Table 5) (44,47-50).

### Route or timing of nutrition interventions

We identified 9 unique studies that examined the route or timing of nutrition interventions initiated before and continued after cancer treatment (Table 4) (54-62). Studies most commonly evaluated adverse events, body weight or composition changes, and length of stay. One study evaluated enteral nutrition immediately initiated after placement of a prophylactic gastrostomy tube (55). A 3-armed trial evaluated changes in the contents and duration of perioperative whole oral immunonutrition (57). Six studies examined the timing of nutrition support in individuals with gastrointestinal cancer, including variation in the timing and/or duration of perioperative nutrition (54,56,58-60,62). Finally, 1 study evaluated consumption of a milk-based oral nutrition supplement (61). These non-US studies took place primarily in Europe and Asia.

### Nutrition support

We identified 13 unique studies across 16 publications examining nutrition support interventions initiated before and continued after the start of cancer treatment (Table 4) (63-78). Studies most commonly evaluated adverse events, length of stay, and survival. Ten studies examined nutrition support in gastrointestinal cancer, using a broad range of nutrition support, including perioperative use of an oral nutrition supplement (69), oral immunonutrition (71), an EPA-enriched supplement (66-68), multi-oil fat emulsion, total parenteral nutrition (63), and whole enteral nutrition in liver cancer (78). The remainder of these 10 studies evaluated use of broader types of nutrition support, including perioperative use of parenteral or enteral nutrition in gastric and colorectal cancer patients (77), peripheral parenteral nutrition in colorectal cancer (74,75), early oral feeding (65), and the use of a preoperative carbohydrate drink (70). The final 3 studies examined peri-treatment nutrition support, including the use of an omega-3 fatty acid-enriched oral nutrition supplement in bladder cancer (73), the use of an amino acid-enriched oral nutrition support in hepatocellular carcinoma (72), and an immune-enhancing diet in head and neck cancer (76). One study was United States based (73), with the majority based in Asia.

Of the 10 studies assessed for RoB (Table 3), 2 low-RoB and 4 medium-RoB studies across 7 publications examined outcomes in surgical inpatients with gastrointestinal cancer (63-65,70,71,74,75). Reported results were mixed, with some studies reporting a benefit and some reporting no difference in improving development of adverse events, length of hospital stay, readmission and emergency room visits, and survival (Table 5).

### Multi-component interventions

One 4-arm study tested a multi-component intervention to improve nutrition, examining the use of dietary advice with nutrition support supplements in England (79).

## Nutrition interventions after treatment began

For KQ2, we identified 141 studies across 150 publications that examined nutrition interventions after cancer treatment began. Studies examined nutrition counseling (n = 15), dietary supplements (n = 34), special diets (n = 8), route or timing of nutrition interventions (n = 31), nutrition support including oral nutrition supplements (n = 43), and multi-component interventions (n = 10) (Table 3).

### Nutrition counseling

We identified 15 unique studies that examined nutrition counseling during cancer treatment (Table 3) (80-94). Studies most commonly included the following outcomes: body weight or composition changes, changes in nutrition status, adverse events, and survival.

Four studies evaluated standardized intensive nutrition counseling across diverse populations, including patients with head and neck cancer undergoing chemoradiotherapy (86), multiple cancer types during and after radiotherapy (92), patients with gastrointestinal and head and neck cancer undergoing radiotherapy (83), and radiation therapy for prostate cancer (82). A second group of studies evaluated individualized nutrition counseling in patients with head and neck cancer undergoing chemoradiotherapy (84), patients with colorectal cancer undergoing radiotherapy (89) and chemotherapy (93), and patients undergoing radiation therapy across multiple cancer types (94). A third group of studies examined face-to-face interviewing among individuals with multiple cancer types undergoing chemotherapy (80,90). The remaining studies were more diverse, evaluating a standardized dietary advice approach for foods to avoid and consume (87); nutrition counseling and meal planning posthospital discharge for multiple cancers; a whole-course nutrition management model (88); the use of individualized dietary planning, nutrition education, and pharmacotherapy; and the use of a motivational interviewing and cognitive behavioral therapy program (81). Most of these studies were conducted in Europe and Asia.

### Dietary supplements

We identified 34 unique studies across 35 publications that examined dietary supplements administered after the start of cancer treatment (Table 4) (95-129). Studies most commonly evaluated body weight or composition changes, adverse events, length of stay, and survival.

Several studies examined the effects of a single supplement, but interventions varied by cancers studied, cancer treatments, length of follow-up, and comparators. Single supplements included the use of arginine-enhanced oral or enteral nutrition support (98,100,102-104,112); omega-3 fatty acids, fish oil, or amino acids (95,99,101,107-109,115,118,119,123,125,126,128,129); glutamine (97,110,113,116,122); EPA (120); and Echium oil (117). A second set of studies examined diverse multi-component supplements across a wide range of cancer populations and treatments, including the use of EPA and gamma-linolenic acid (114); triglycerides and protein (124); arginine, omega-3 fatty acids, and RNA (105); arginine, glutamine, and cysteine (111); protein and arginine (121); arginine, glutamine, and fish oil (96); EPA and docosahexaenoic acid (106); and omega-3 fatty acid, glutamine, and probiotics (127). Studies in dietary supplements were predominately conducted within Europe and Asia.

Among the 26 studies assessed for RoB (Table 3; 4 low-RoB and 14 medium-RoB studies), results were mixed, with most studies reporting no benefit of added dietary supplements on body weight, adverse events, length of hospital stay, or survival (Table 5) (100-103,105,107,109,111,113,115-119,123,124,126,129).

### Special diets

We identified 8 unique studies across 9 publications that examined special diets after the initiation of cancer treatment (Table 4) (130-138). Studies evaluated changes in body weight or composition and adverse events. Two studies evaluated the use of special drinks, including wine (132) or grape juice (131) before meals. Three studies evaluated fasting or calorie restriction, including calorie-restricted ketogenic diets (133,134,137) or a fasting-mimicking diet (135). The remaining studies examined modified diets by consistency or contents, including early initiation of a solid vs liquid diet after surgery (136), a diet containing no raw fruits or vegetables (130), and a low-fat or modified-fat diet (138). Studies were predominantly conducted in North America and Europe.

### Route or timing of nutrition interventions

We identified 31 unique studies that examined the route or timing of nutrition interventions delivered, at least in part, during cancer treatment (Table 4) (139-169). Studies most commonly evaluated adverse events and length of stay. Seventeen studies compared enteral nutrition vs parenteral nutrition, predominately in postoperative individuals (140-142,146-153,156,158,164,166-168). Six studies compared oral feeding vs enteral or parenteral methods (139,144,145,157,160,162). Three studies evaluated the effects of different enteral or parenteral methods, including use of percutaneous endoscopic gastrostomy tube vs a nasogastric tube (159), jejunostomy feeding vs nasogastric feeding (163), and use of a peripherally inserted central catheter for administration of parenteral nutrition vs a central venous catheter (169). Finally, 5 studies examined variation in the timing of oral feeding after cancer surgery (143,154,155,161,165). Studies were predominately conducted in Asia and Europe.

Among 24 studies assessed for RoB (Table 3), 1 low-RoB and 10 medium-RoB studies that examined route and timing of nutrition interventions conducted during cancer treatment demonstrated mixed results (139,141,142,144,149,153,162,163,165-167). The majority reported no difference for body weight, adverse events, readmissions, or death, but one-half reported reduced length of hospital stay (Table 5).

### Nutrition support

We identified 43 unique studies across 49 publications that examined nutrition support interventions during cancer treatment (Table 4) (170-218). Studies most commonly evaluated adverse events and body weight or composition changes.

Twenty-one studies from 26 publications examined oral or enteral nutrition support after cancer surgery, and they varied in the route and contents of nutrition support delivered as well as the timing of the comparisons. Specifically, several studies examined immunonutrition after surgery, and another compared early enteral nutrition vs parenteral nutrition (200). Among the remaining studies, the nutrition support varied in contents and quantity, including nutrition support supplements such as Elental (182,183), Jevity (193), Racol (199), Nutren (197,198), and Nutrison Fibre (214), and other enteral nutrition formulations across diverse cancer types after surgery (171,172,175,176,178,180,212,215,217). Finally, 1 distinct study evaluated the use of iEAT, food disintegrated on the tongue, vs standard care after surgery for gastrointestinal cancer (207).

Seventeen studies from 18 publications examined oral or enteral nutrition support during chemotherapy or radiation, and they varied in the route and contents of nutrition support delivered as well as the timing of comparisons. Three studies examined EPA-enriched oral nutrition support (170,186,205). Another 3 studies examined hyper-protein and omega-3 fatty acid-enhanced nutrition support (174,179,218). The remaining studies evaluated standard nutrition support across diverse populations and intervention dose.

Finally, 4 studies examined total parenteral nutrition (TPN) across cancer and treatment types (173,185,209,211), with 1 additional study examining combined enteral and parenteral nutrition after esophageal cancer surgery (177). Among 27 studies assessed for RoB (Table 3), 3 low-RoB and 8 medium-RoB studies evaluated the use of nutrition support during cancer treatment (172,177,188-192,194,197-199,211,212,214,217). Two low- (197,217), and 3 medium-RoB studies (199,211,212) reported improvements in body weight or composition with postoperative nutrition support. Four out of 10 studies (172,177,194,214) reported improvements in adverse events and 3 reported reductions in length of hospital stay (172,177,214) across diverse enteral and oral nutrition support interventions.

### Multi-component interventions

Ten studies across 11 publications examined multi-component interventions initiated after cancer treatment began (Table 4) (219-229). Studies most commonly evaluated body weight or composition changes as well as changes in nutrition status.

Multi-component interventions varied in the interventions administered, cancer type, comparators, and cancer treatments. Four studies examined individualized diet or nutrition plans combined with additional interventions such as education or counseling (219,223,228,229). The remaining studies widely varied in interventions administered, including the use of individual recipes developed by patients, caregivers, and clinical specialists (221); a calcium-rich, low-fat and high-fruit and vegetable diet plus exercise (220); prophylactic percutaneous endoscopic gastrostomy tube along with nutrition advice (224,225); oral, enteral, or TPN nutrition followed by nutrition education; and in-hospital nutrition education and early enteral nutrition support (227).

## Effect of nutrition interventions on symptoms

For KQ3, we identified 79 studies across 83 publications that examined nutrition interventions before or during cancer treatment on symptoms. Studies examined nutrition counseling (n = 12), dietary supplements (n = 10), special diets (n = 10), route or timing of nutrition interventions (n = 8), nutrition support (including oral nutrition supplements) (n = 32), and multi-component interventions (n = 7) (Table 3).

### Nutrition counseling

We identified 12 unique studies examining nutrition counseling on improving cancer symptoms before or during cancer treatment (Table 4) (81-83,85,87-90,92-94,230). Studies most commonly included the following outcomes: quality of life, symptoms, and functional status. Three studies evaluated standardized intensive nutrition counseling (82,83,92). Another group of studies evaluated individualized nutrition counseling or face-to-face interviewing (89,90,93,94,230). The remaining studies were more diverse, including standardized dietary advice for foods to avoid or consume (87), a whole-course nutrition management model (88), individualized dietary planning plus nutrition education and pharmacotherapy, and use of motivational interviewing and cognitive behavioral therapy (81). Most studies were conducted in Europe and Asia.

### Dietary supplements

We identified 10 unique studies that examined dietary supplements initiated before or during cancer treatment and evaluated the impact of the intervention on symptoms (18,39,47,51,106-108,120,122,124). Studies most commonly evaluated quality of life and symptoms.

Seven studies examined a single supplement in a variety of populations with a wide range of durations and treatment contexts, including use of omega-3 fatty acids (107), EPA-enriched supplements (106), orally administered amino acid jelly (108), glutamine injection, oral whey protein supplementation (39), and amino acid supplements (51). One 5-arm study evaluated the combinations of EPA, l-carnitine, thalidomide, and medroxyprogesterone acetate or megestrol acetate. Three studies examined multiple supplements to improve cancer treatment–related symptoms, including oral protein supplementation rich in arginine and omega-6 (47); fatty acids, arginine, fiber, and nucleotides (18); and medium-chain triglycerides and protein (124).

Of the 5 studies assessed for RoB (Table 3), 3 medium-RoB studies in dietary supplements reported mixed results for patient-reported symptoms (47,107,124). One study of probiotics and omega-3 fatty acids reported improved quality of life (107), whereas another reported no benefit (47). Two studies reported mixed outcomes for patient-reported symptoms, with 1 reporting benefit (107) and the other reporting no difference (Table 5) (124).

### Special diets

We identified 10 unique studies across 11 publications that examined the effects of special diets before or during cancer treatment on treatment-related symptoms (130-138,231,232). Studies most commonly evaluated quality of life and symptoms.

Two studies evaluated the use of specific drinks, with 1 evaluating consumption of a glass of wine (132) or grape juice (131) before meals. Another 4 studies examined fasting or calorie restriction, including a calorie-restricted ketogenic diet (133,134,137), a fasting-mimicking diet (135), and calorie restriction with synbiotics (232). The remaining studies examined modified diets by consistency or contents, including evaluation of early initiation of a solid diet (136), a diet containing no raw fruits or vegetables (130), ginger (231), and a low or modified-fat diet (138). Most of these studies were conducted in North America, Europe, and Asia.

### Route or timing of nutrition intervention

We identified 8 studies that examined whether the route or timing of nutrition interventions delivered, at least in part, during cancer treatment affected cancer or cancer treatment-related symptoms, with most studies evaluating quality of life (55,60,61,144,157,159,162,163).

Of the 8 studies, 4 compared early oral feeding vs enteral nutrition. Another evaluated timing of delivery of a milk-based oral nutrition supplement (61). The remaining studies were more varied but focused on evaluating the mode of nutrition interventions on symptoms of cancer treatment, including the use of a percutaneous endoscopic gastrostomy tube vs a nasogastric tube (159), the use of jejunostomy feeding or nasogastric feeding (163), and total parenteral nutrition vs an oral diet (157).

### Nutrition support

We identified 32 studies across 34 publications that examined the effect of nutrition support interventions during cancer treatment on cancer treatment-related symptoms (25,27,29,32,34,65,69,72,76,171,173,174,176,179-181,184-186,195,197,198,202-205,208-213,217,218).

Fifteen studies examined oral or enteral nutrition support before or after cancer surgery; the studies varied in the route and contents of nutrition support delivered and the timing of the intervention and comparison groups. Thirteen studies examined oral or enteral nutrition support before or after chemotherapy and/or radiation; however, they also considerably varied in the route and contents of nutrition support delivered, populations, and timing of the intervention and comparison groups (72,89,174,179,181,184,186,195,205,208,210,213,218). Finally, 4 studies examined TPN across cancer and treatment types (173,185,209,211).

Of the 20 studies assessed for RoB (Table 3), 5 low-RoB and 4 medium-RoB studies of nutrition support reported mixed results (13,15,16,18,28,66,68,69,71,72). Two studies showed mixed results on functional status, with 1 showing a benefit and the other reporting no difference (28,66). Two low-RoB studies reported improvement in nausea for individuals receiving preoperative oral carbohydrate drinks (28,66). A third low-RoB study reported improvement in treatment tolerance and symptoms after use of oral nutrition supplements and dietary advice (Table 5) (66,68).

### Multi-component interventions

Seven studies across 8 publications examined multi-component nutrition interventions administered before or during cancer treatment. Two studies examined individualized diet plans combined with interventions such as education or counseling (222,228). The remaining studies varied in the types of interventions delivered, including evaluation of a calcium-rich, low-fat, and high-fruit-and-vegetable diet (220); dietary advice plus a supplement (79); in-hospital nutrition education and early enteral nutrition support; avoidance of food high in dietary fiber and lactose (233); and a prophylactic percutaneous endoscopic gastrostomy tube along with nutrition advice (224,225).

## Variation in the effect of nutrition interventions (KQs 1-3)

Although studies addressing KQ1-3 enrolled varied samples (eg, cancer type and stage, treatment, age, comorbid conditions, and degree of muscle wasting), no eligible studies specifically evaluated whether the effects of nutrition interventions on preventing negative outcomes varied across these characteristics.

## Effect of nutrition interventions on weight loss

Four studies reported the effects of nutrition interventions intended for body weight loss for overweight or obesity using special diets among individuals with breast cancer (Table 4). Studies varied in the enrolled populations, interventions. and comparison groups. Studies included evaluation of the effect of calorie restriction with synbiotics (232), a diet designed to prevent weight gain, the use of a Mediterranean diet and dietary advice, and intermittent 25% energy restriction. Studies were conducted predominantly in Europe and Asia.

## Cost or value of nutrition interventions

Among studies evaluating the effectiveness of nutrition interventions included in our KQs, few (8/206) published any cost or value (eg, cost-effectiveness, cost-benefit) information related to the intervention. Most often, the cost-related information focused on the total cost of care for 1 group vs a comparison group, making it challenging to identify the exact cost of the intervention or its components. In a grey literature search (Supplementary Materials), we identified a few additional studies that examined the cost or value of nutrition interventions, including examination of the cost-effectiveness of preoperative immunonutrition (234,235), perioperative enteral nutrition in colorectal cancer, supplemental parenteral nutrition for inoperable pancreatic cancer (236), and a value analysis of nutrition interventions for gastrointestinal cancer. These studies were predominantly conducted in inpatient settings in non-US health systems.

## Discussion

Two decades of randomized trial evidence on nutrition interventions for adults prior and/or during cancer treatment provide only limited high-quality evidence to inform supplemental nutrition recommendations that could improve cancer treatment-related outcomes.

The questions of whether nutrition interventions (or components) can prevent negative health outcomes, for whom, and under what circumstances are of vital importance to patients and clinicians. The number of individuals at risk for cancer-related malnutrition remains substantial, with estimates ranging from 25% to 80% across patient populations (7-9). Because cancer risk (and malnutrition) increases with age, the rapidly growing older population in the United States will increase the demand for cancer care and, by extension, nutrition therapy over the coming decades (237).

There are sizeable limitations in the evidence base on nutrition interventions in adult cancer care. The topic of nutrition interventions to prevent adverse effects of treatments for cancer patients at risk for malnutrition is extremely broad. This breadth creates challenges for adequately categorizing and summarizing clinical evidence. Although we identified many studies, they widely differed by population, intervention type, intent, timing, mode of delivery, comparators, cancer types, treatments, and outcomes. Even within intervention categories, we found substantial heterogeneity, making aggregation infeasible. Furthermore, definitions of malnutrition (or at risk for) widely varied, and many interventions (eg, dietary supplements) are unlikely to improve malnutrition outcomes such as weight loss in the time frame of the studies. Additionally, most studies were small and of a short duration.

Based on our systematic review, we note several potential areas to target to improve research strategies in cancer-associated malnutrition to better inform clinical practice and policy. First, the literature broadly lacked a clear conceptual framework describing how each intervention would be expected to improve outcomes. As a result, the field could benefit from development of a comprehensive and clear conceptual framework addressing how specific nutrition interventions could improve the key outcomes most important to stakeholders. Second, we were struck by the lack of adherence to basic reporting standards within these publications, including missing information on the random assignment process, blinding of participants and assessors, and populations analyzed. Future RCT research on nutrition interventions should emphasize consistent use of criteria laid out in the CONSORT statement (238).

Third, although clinical stakeholders were eager to understand the available literature within specific populations of individuals receiving nutrition interventions (eg, adults ≥65 years old, those with muscle wasting, individuals with comorbid conditions), studies rarely reported results according to these characteristics. Rather, studies tended to focus on very specific populations (eg, early-stage gastric cancer), and almost all were conducted outside of the United States, mainly in Asia and Europe, where generalizability of nutrition norms, costs of care, and available resources differ greatly from the United States.

Fourth, definitions of malnutrition widely varied. Although weight loss is one common definition, many studies enrolled individuals considered malnourished based on biochemical or micronutrient abnormalities rather than weight. Furthermore, many individuals with localized cancer may be malnourished for reasons not due to cancer.

Fifth, we noted considerable diversity in the nutrition interventions tested, where outcomes were focused on the reduction of the negative effects of cancer treatment and associated symptoms. Nutrition interventions were extremely heterogeneous, even within intervention types. For example, studies using dietary supplements included omega-3 fatty acids, glutamine, or arginine, among others. Even when the studies used the same interventions, they varied considerably in the populations studied, duration (eg, 2 days vs 3 months), dosage, route, and type of cancer treatment. Heterogeneity in intervention type and reporting, as well as the absence of key information, limited our ability to conduct meta-analyses.

Sixth, outcomes collected to assess the impact of nutrition interventions were diverse and ranged from intermediate outcomes, such as changes in body weight, to longer-term outcomes, such as changes in overall survival. Outcomes were often poorly defined, with few details provided on the timing, definitions, or evaluation of outcomes (how, by whom, over what time frame, and using what criteria). One crucial way to improve outcome assessment in future nutrition intervention studies is to use standardized assessments with common, validated tools coupled with clearly defined assessment time periods that are consistent between the intervention and comparison group.

Finally, we focused our report on individuals judged to have physiologic or biochemical measures of, or at risk for, malnutrition. We did not evaluate interventions specifically targeted to individuals at risk for malnutrition due to sociodemographic factors, including food insecurity and healthy dietary choices. To better inform future implementation, additional research should examine the effects of interventions across populations and ensure they are applicable to future real-world implementation.

We acknowledge the following limitations of the review. We used broad definitions of nutrition interventions, thereby increasing the scope, breadth, and heterogeneity of the included literature to better assess the range and depth of available evidence. This decision allowed for a demonstration of the diffuse literature set on the topic and highlighted the predominantly low quality of the literature where there were concentrations of similar intervention types. However, this required focusing on high-level directionality of intervention effects across a broader range of nutrition interventions rather than detailed, precise estimates of intervention effects. Nonetheless, we failed to find strong signals indicating clear, consistent benefits of nutrition interventions in any populations or cancer therapies. Our review did not assess the long-term effects of nutrition interventions on cancer outcomes including cancer control and cancer-specific mortality. We presumed that nutrition interventions that improved effective cancer treatment initiation, adherence, and tolerance and reduced treatment-related adverse effects would lead to improved long-term cancer-related outcomes.

Overall, these findings should serve to encourage and suggest ways to bolster the rigor and reporting of future research to inform clinical practice and guideline development on nutrition interventions for cancer. Future investigation of the role of malnutrition screening would fill an important gap but was not addressed by this review. Another important future direction would be investigations to optimize and standardize methods for widespread nutrition assessment and intervention implementation in clinical settings. Future funding efforts, which should align with the priorities of the National Institutes of Health (NIH) Precision Nutrition Initiative (239), include 1) standardizing definitions and taxonomies for populations, interventions, and outcomes, including identifying those with and at risk for malnutrition; 2) improving rigor in the primary intent, design, and reporting of nutrition interventions; and 3) coordinating efforts to develop detailed conceptual frameworks for mechanisms of nutrition interventions effects across patient nutrition risk categories, cancers, and cancer treatments. These efforts will help prioritize research agendas as well as inform study designs and, ultimately, clinical practice and health policy.

## Supplementary Material

pkad035_Supplementary_Data

## Data Availability

The data underlying this article are available in the article and in its online supplementary material.
