# Supplementary material for: Nutrition as prevention for improved cancer health outcomes: a systematic literature review
Source: JNCI Cancer Spectr. 2023 May 22;7(3):pkad035. doi: 10.1093/jncics/pkad035 (PMC10290234; doi:10.1093/jncics/pkad035)
Supplement: pkad035_Supplementary_Data [file pkad035_supplementary_data.pdf]

Supplementary Materials: Nutrition as Prevention for Improved Cancer Health Outcomes
Search Strategy

The search strategy was designed and conducted by an experienced systematic review/medical librarian with input from the investigators. We applied the following limits or filters to the database searches:

- Date. We considered a literature search starting in 2000 sufficient for the purpose of this review. Date limitation was done in PICO Portal.
- Language. Publications were excluded if they were written in a language other than English. This was due to resource constraints.
- Publication status. We searched for published studies.
- Humans or organisms. A filter was used to remove animal studies.
- Study Design. The search was limited to randomized controlled trials, controlled trials, and observational cohort studies. After review of the breadth of included studies, study designs were further limited to randomized controlled trials randomizing at least 50 participants (i.e., 25 individuals per arm) to identify the literature with the highest likelihood of having statistical power to detect an effect from a nutrition intervention. The updated search on July 22, 2022 was limited to randomized controlled trials.

We conducted a comprehensive literature search in May 2021 (updated July 2022). We searched the following databases:

- Ovid MEDLINE(R) and Epub Ahead of Print, In-Process, In-Data-Review & Other Non-Indexed Citations, Daily and Versions(R) <1946 to May 21, 2021> Date searched: May 20, 2021
- Cochrane Central Register of Controlled Trials (Wiley) Issue 4 of 12, April 2021 Date searched: May 21,2021
- Embase Classic+Embase <1947 to 2021 May 20> (Ovid) Date searched: May 20, 2021.

For the contextual question we conducted a literature search through July 2022 to more broadly understand the effectiveness of nutrition interventions from a cost-perspective, including evaluations that reported information on intervention costs as well as cost-effectiveness, cost-benefit and value analyses. We searched the following databases:

- References of relevant systematic reviews and meta analysis (line 35 of Ovid MEDLINE search strategy below)
- Web search engines/specific web sites. We searched for grey literature using Google. The first 200 Google results yielded by each search string were reviewed.
- Search of the Center for the Evaluation of Value and Risk in Health, Tufts Cost-Effectiveness Analysis Registry

Database search strategies

Updated Search: Ovid MEDLINE(R) ALL <1946 to July 22, 2022>

1 Nutrition Therapy/ or Diet Therapy/ or ((diet* or nutrition*) adj3 (counsel* or intervention* or support* or supplement* or therap*)).ti,ab. 1
2 Prebiotics/ or Probiotics/ or Synbiotics/ or (prebiotic* or probiotic* or symbiotic* or synbiotic*).ti,ab.
3 Enteral Nutrition/ or Nutritional Support/ or exp Parenteral Nutrition/ or ((enteral or gastrostomy or jejunostomy or oral or parenteral or tube) adj3 (feeding or nutrition*)).ti,ab.
4 Caloric Restriction/ or Diet, Reducing/ or (calori* restrict* diet* or intermittent fasting or fasting mimicking diet* or short-term fasting).ti,ab.
5 Diet, High-Protein/ or Diet, Ketogenic/ or Diet, Carbohydrate-Restricted/ or Diet, High-Protein Low-Carbohydrate/ or Diet, Mediterranean/ or (high-protein diet* or high-calorie diet* or ketogenic diet* or mediterranean diet*).ti,ab.
6 or/1-5
7 Brachytherapy/ or Chemoprevention/ or Chemoradiotherapy/ or Chemoradiotherapy, Adjuvant/ or Chemotherapy, Adjuvant/ or Consolidation Chemotherapy/ or exp Neoplasms/ or Radiotherapy/ or (cancer* or carcinoma* or chemoprevention or chemotherap* or chemoradiotherap* or leuk?emia* or melanoma* or myeloma* or neoplasm* or radiotherap* or radiation therap*).ti,ab.
8 6 and 7
9 control groups/ or double-blind method/ or placebo effect/ or random allocation/ or exp randomized controlled trial/ or single-blind method/
10 (control* adj3 (study or studies or trial* or group*)).ti,ab,hw,kf.
11 (random* or sham or placebo*).ti,ab,hw,kf.
12 ((singl* or doubl*) adj (blind* or dumm* or mask*)).ti,ab,hw,kf.
13 ((tripl* or trebl*) adj (blind* or dumm* or mask*)).ti,ab,hw,kf.
14 or/9-13
15 8 and 14
16 limit 15 to english language
17 limit 16 to dt=20210501-20220731

Database: Embase <1974 to 2022 Week 29>

1 diet therapy/ or nutritional counseling/ or diet supplementation/ or ((diet* or nutrition*) adj3 (counsel* or intervention* or support* or supplement* or prebiotic* or probiotic* or symbiotic* or synbiotic* or therap*)).ti,ab. Or
2 nutritional support/ or parenteral nutrition/ or enteric feeding/ or ((enteral or gastrostomy or jejunostomy or oral or parenteral or tube) adj3 (feeding or nutrition*)).ti,ab.
3 (malnutrition universal screening or malnutrition screening or nutrition* assessment or nutrition* risk screening).ti,ab.
4 caloric restriction/ or intermittent fasting/ or exp low calorie diet/ or (calori* restrict* diet* or intermittent fasting or fasting mimicking diet* or short-term fasting).ti,ab.
5 exp ketogenic diet/ or exp protein diet/ or Mediterranean diet/ or low carbohydrate diet/ or (high-protein diet* or high-calorie diet* or ketogenic diet or mediterranean diet).ti,ab.
6 or/1-5
7 exp neoplasms subdivided by anatomical site/ or cancer chemotherapy/ or chemoprophylaxis/ or consolidation chemotherapy/ or cancer radiotherapy/ or brachytherapy/ or chemoradiotherapy/

or adjuvant chemoradiotherapy/ (cancer* or carcinoma* or chemoprevention or chemotherap* or chemoradiotherap* or leuk?emia* or melanoma* or myeloma* or neoplasm* or radiotherap* or radiation therap*).ti,ab.
8 6 and 7
9 (rats or rat or rabbit or porcine or cow or cows or chicken* or horse or horses or mice or mouse or bovine or sheep or ovine or murinae or cats or cat or dog or dogs or rodent or swine).tw.
10 8 not 9
11 exp randomized controlled trial/ or controlled clinical trial/ or control group/ or double blind procedure/ or randomization/ or single blind procedure/ or placebo effect/
12 (control* adj3 (study or studies or trial* or group*)).ti,ab,kw.
13 (random* or sham or placebo*).ti,ab,kw.
14 ((quasiexperimental or quasi experimental) adj3 (study or studies or trial*)).ti,ab,kw.
15 or/11-14
16 10 and 15
17 limit 16 to dd=20220701-20220731
18 limit 17 to conference abstracts
19 17 not 18

Cochrane Central Register of Controlled Trials Date Run: 07/31/2022
Issue 7 of 12, July 2022

ID Search Hits
#1 [mh ^"diet therapy"] or [mh ^"nutrition therapy"]
#2 (diet NEAR/3 (counsel or intervention or support or supplement or therapy)):ti,ab (Word variations have been searched)
#3 (nutrition NEAR/3 (counsel or intervention or support or supplement or therapy)):ti,ab (Word variations have been searched)
#4 [mh prebiotics] or [mh probiotics] or [mh symbiotics] or [mh synbiotics]
#5 (prebiotic? or probiotic? or symbiotic? or synbiotic?):ti,ab
#6 [mh "nutritional support"] or [mh "enteral nutrition"] or [mh "parenteral nutrition"]
#7 enteral:ti,ab or gastrostomy:ti,ab or jejunostomy:ti,ab or parenteral:ti,ab or (tube near/2 feeding):ti,ab
#8 [mh "caloric restriction"] or [mh "diet, reducing"]
#9 (calorie near/3 restrict):ti,ab or (intermittent near/2 fasting):ti,ab or (fasting near/2 mimicking):ti,ab or ("short term" near/2 fasting):ti,ab (Word variations have been searched)
#10 [mh "Diet, High-Protein"] or [mh "diet, ketogenic"] or [mh "diet, carbohydrate-restricted"] or [mh "diet, high-protein low-carbohydrate"] or [mh "diet, mediterranean"]
#11 ("high protein" near/3 diet):ti,ab or ("high calorie" near/3 diet):ti,ab or (ketogenic near/3 diet):ti,ab or (Mediterranean near/3 diet):ti,ab (Word variations have been searched)
#12 [OR #1-#11]
#13 [mh Neoplasms] or [mh chemoprevention] or [mh chemoradiotherapy] or [mh chemoradiotherapy] or [mh "chemoradiotherapy, adjuvant"] or [mh "chemotherapy, adjuvant"] or [mh "consolidation chemotherapy"] or [mh ^radiotherapy] or [mh brachytherapy]
#14 (cancer or carcinoma or chemoprevention or chemotherapy or chemoradiotherapy or

leukemia or melanoma or myeloma or neoplasm or radiotherapy or "radiation therapy" or "radiation therapies"):ti,ab (Word variations have been searched)
#15 #13 or #14 235400
#16 #12 and #15 in Trials 4965
2022-05-01 to 2022-07-31: 25 total including 8 clinical trials from clinicatrials.gov

Original Searches May 2021

Ovid MEDLINE(R) and Epub Ahead of Print, In-Process, In-Data-Review & Other Non-Indexed Citations, Daily and Versions(R) <1946 to May 21, 2021>

1 Nutrition Therapy/ or diet therapy/ or ((diet* or nutrition*) adj3 (counsel* or intervention* or support* or supplement* or therap*)).ti,ab.
2 Prebiotics/ or Probiotics/ or Synbiotics/ or (prebiotic* or probiotic* or symbiotic* or synbiotic*).ti,ab.
3 Enteral Nutrition/ or Nutritional Support/ or exp Parenteral Nutrition/ or ((enteral or gastrostomy or jejunostomy or oral or parenteral or tube) adj3 (feeding or nutrition*)).ti,ab.
4 Caloric Restriction/ or Diet, Reducing/ or (calori* restrict* diet* or intermittent fasting or fasting mimicking diet* or short-term fasting).ti,ab.
5 Diet, High-Protein/ or Diet, Ketogenic/ or Diet, Carbohydrate-Restricted/ or Diet, High-Protein Low-Carbohydrate/ or Diet, Mediterranean or (high-protein diet* or high-calorie diet* or ketogenic diet* or mediterranean diet*).ti,ab. or /
6 or/1-5
7 Brachytherapy/ or Chemoprevention/ or Chemoradiotherapy/ or Chemoradiotherapy, Adjuvant/ or Chemotherapy, Adjuvant/ or Consolidation Chemotherapy/ or exp Neoplasms/ or Radiotherapy/ or (cancer* or carcinoma* or chemoprevention or chemotherap* or chemoradiotherap* or leuk?emia* or melanoma* or myeloma* or neoplasm* or radiotherap* or radiation therap*).ti,ab.
8 Controlled Clinical Trial/ or Control Groups/ or Double-Blind Method/ or Placebo Effect/ or exp Randomized Controlled Trial/ or Random Allocation/ or Single-Blind Method/
9 (control* adj3 (study or studies or trial* or group*)).ti,ab,hw,kf.
10 (random* or sham or placebo*).ti,ab,hw,kf.
11 ((quasiexperimental or quasi experimental) adj3 (study or studies or trial*)).ti,ab,hw,kf.
12 (nonrandom* or non random* or quasi-random* or quasirandom*).ti,ab,hw,kf.
13 or/8-12
14 Epidemiologic Studies/
15 exp Case-Control Studies/
16 exp Cohort Studies/
17 Cross-Sectional Studies/
18 (epidemiologic adj (study or studies)).ti,ab.
19 case control*.ab,ti.
20 (cohort adj3 (study or studies)).ti,ab.
21 cross sectional.ab,ti.
22 cohort analy*.ab,ti.
23 (follow up adj3 (study or studies)).ti,ab.

24 longitudinal.ti,ab.
25 retrospective*.ti,ab.
26 prospective*.ti,ab.
27 (observ* adj3 (study or studies)).ti,ab.
28 or/14-27
29 13 or 28
30 6 and 7 and 29
31 (rats or rat or rabbits or rabbit or porcine or cow or cows or chicken* or horse or horses or mice or mouse or bovine or sheep or ovine or murinae or cats or cat or dog or dogs or rodent or swine or pigs or pig).tw.
32 30 not 31
33 limit 32 to (comment or editorial or letter or news or newspaper article or personal narrative or preprint)
34 32 not 33
35 limit 34 to (meta analysis or "systematic review")
36 34 not 35
37 limit 36 to english language

Embase Classic+Embase <1947 to 2021 May 20> (Ovid)

1 diet therapy/ or nutritional counseling/ or ((diet* or nutrition*) adj3 (counsel* or intervention* or support* or supplement* or therap*)).ti,ab.
2 enteric feeding/ or nutritional support/ or parenteral nutrition/ or ((enteric or enteral or gastrostomy or jejunostomy or oral or parenteral or tube) adj3 (feeding or nutrition*)).ti,ab.
3 diet supplementation/ or prebiotic agent/ or probiotic agent/ or synbiotic agent/ or (prebiotic* or probiotic* or symbiotic* or synbiotic*).ti,ab.
4 exp diet restriction/ or (calori* restrict* diet* or intermittent fasting or fasting mimicking diet* or short-term fasting).ti,ab.
5 low carbohydrate diet/ or exp ketogenic diet/ or exp protein diet/ or Mediterranean diet/ or (high-protein diet* or high-calorie diet* or ketogenic diet or mediterranean diet).ti,ab.
6 1 or 2 or 4 or 5
7 adjuvant chemoradiotherapy/ or brachytherapy/ or cancer chemotherapy/ or cancer radiotherapy/ or chemoprophylaxis/ or chemoradiotherapy/ or consolidation chemotherapy/ or exp malignant neoplasms/ or (cancer* or carcinoma* or chemoprevention or chemotherap* or chemoradiotherap* or leuk?emia* or melanoma* or myeloma* or neoplasm* or radiotherap* or radiation therap* or tumo?r*).ti,ab.
8 6 and 7
9 (rats or rat or rabbits or rabbit or porcine or cow or cows or chicken* or horse or horses or mice or mouse or bovine or sheep or ovine or murinae or cats or cat or dog or dogs or rodent or swine or pig or pigs).tw.
10 8 not 9
11 controlled clinical trial/ or control group/ or double blind procedure/ or placebo effect/ or exp randomized controlled trial/ or randomization/ or single blind procedure/
12 (control* adj3 (group* or study or studies or trial*)).ti,ab,kw.
13 (placebo or random* or sham).ti,ab,kw.
14 ((quasiexperimental or quasi experimental) adj3 (study or studies or trial*)).ti,ab,kw.

15 or/11-14
16 10 and 15
17 epidemiology/ or epidemiolog* study.ti,ab.
18 cross-sectional study/ or cross sectional.ti,ab,kw.
19 cohort analysis/
20 case control study/
21 observational study/
22 prospective study/
23 longitudinal study/
24 retrospective study/
25 case control*.ti,ab,kw.
26 (cohort adj3 (study or studies)).ti,ab,kw.
27 cross sectional.ti,ab,kw.
28 cohort analy*.ti,ab,kw.
29 (longitudinal or prospective* or retrospective*).ti,ab,kw.
30 (observ* adj3 (study or studies)).ti,ab,kw.
31 or/17-30
32 15 or 31
33 10 and 33
34 limit 34 to english language
35 limit 34 to (conference abstract or conference paper or "conference review" or editorial or letter or note or "review")
36 34 not 35
37 limit 36 to (meta analysis or "systematic review")
38 36 not 37

Cochrane Central Register of Controlled Trials (Wiley)
Issue 5 of 12, May 2021

ID Search Hits
#1 [mh ^"diet therapy"] or [mh ^"nutrition therapy"]
#2 (diet* NEAR/3 (counsel* or intervention* or support* or supplement* or therap*)):ti,ab
#3 (nutrition* NEAR/3 (counsel* or intervention* or support* or supplement* or therap*)):ti,ab
#4 [mh prebiotics] or [mh probiotics] or [mh symbiotics] or [mh synbiotics]
#5 (prebiotic* or probiotic* or symbiotic* or synbiotic*):ti,ab
#6 [mh "nutritional support"] or [mh "enteral nutrition"] or [mh "parenteral nutrition"]
#7 (enteral or gastrostomy or jejunostomy or parenteral or tube feeding):ti,ab
#8 [mh "caloric restriction"] or [mh "diet, reducing"]
#9 (calori* NEAR restrict* or intermittent NEAR fasting or fasting NEAR mimicking or short-term NEAR fasting):ti,ab
#10 [mh "Diet, High-Protein"] or [mh "diet, ketogenic"] or [mh "diet, carbohydrate-restricted"] or [mh "diet, high-protein low-carbohydrate"] or [mh "diet, mediterranean"]
#11 (high-protein diet* or high-calorie diet* or ketogenic diet or mediterranean diet):ti,ab
#12 [OR #1-#11]

#13 [mh brachytherapy] or [mh chemoprevention] or [mh chemoradiotherapy] or [mh chemoradiotherapy] or [mh "chemoradiotherapy, adjuvant"] or [mh "chemotherapy, adjuvant"] or [mh "consolidation chemotherapy"] or [mh Neoplasms] or [mh ^radiotherapy]
#14 (cancer* or carcinoma* or chemoprevention or chemotherap* or chemoradiotherap* or leuk?emia* or melanoma* or myeloma* or neoplasm* or radiotherap* or radiation therap*):ti,ab
#15 #13 or #14
#16 #12 and #15 3647 (PubMed, Embase, CINAHL)

Tufts CEA Registry Search Terms

“diet therapy" or “nutrition therapy" or “counsel*” or “nutrition supplement*” or “enteral nutrition” or “parenteral nutrition” or “oral nutrition supplement” or “caloric restriction” or “diet” AND “cancer” or “carcinoma”
